# Solvent effect on two-photon absorption and fluorescence of rhodamine dyes

**DOI:** 10.1016/j.jphotochem.2009.06.007

**Published:** 2009-08-15

**Authors:** Amit Nag, Debabrata Goswami

**Affiliations:** Department of Chemistry, Indian Institute of Technology, Sl-216, Kanpur 208016, Uttar Pradesh, India

**Keywords:** TPA, TPF, Solvent polarity, Vibronic coupling, Figure-of-merit

## Abstract

For a series of rhodamine dyes, two-photon absorption (TPA) and two-photon fluorescence (TPF) have been performed in different solvents. Solvent-dependent TPA spectra of these dyes were measured with open aperture z-scan method and compared to their respective single-photon spectra at equivalent energies. In the TPA spectra, relative peak intensities and positions are highly solvent dependent, which could be a result of vibronic couplings that depend on solvent environment. Measured TPA cross-sections of rhodamine dyes are consistently higher in nonpolar solvents. Certain complementary and similarity between TPA and TPF are also elucidated. Finally, a two-photon figure-of-merit is presented for these dyes in different solvents as a function of wavelength.

## 1 Introduction

Over the past one and half decades, study of two-photon absorption (TPA) [1] of molecules have pioneered technologies, such as, two-photon imaging microscopy [2], photodynamic therapy [3], optical limiting [4], all-optical switching and signal processing [5], etc. For such applications, design and synthesis of molecules with large TPA cross-section (TPCS) values has received a lot of attention [6–8]. Using femtosecond lasers, we have accurately measured TPCS (*σ*_2_) values with high sensitivity for various chromophores [9–13]. Though molecular design has been the most dominant scheme for achieving large *σ*_2_ values, there have also been several indications [14–20] that effect of solvent polarity can play an important role in the modulation of *σ*_2_ of the chromophores. However, there is a lack of systematic study in this direction. In fact, the effect of solvent polarity on TPA is not well understood yet. Extensive theoretical and experimental research has been devoted to understand the solvent dependence of linear spectroscopy [21–23] but similar efforts to address solvent effects on TPA of conjugated molecules have been limited. Theoretical studies on push-pull molecule [14,17] show strong dependence of the molecular geometry in solution associated with the polarities of the solvents, and thus, polar solvents lead to bigger TPCS. However, solvent effect studies on the TPA of *D*–π–*A* systems [15] displayed a nonmonotonic behavior of TPCS with respect to the solvent polarity, though there is an enhancement of the *σ*_2_ in solution. Other theoretical studies with more sophisticated models [16], have found that the TPCS of a charge-transfer molecule is more dependent on the optical dielectric constant rather than the static dielectric constant of the solvent. Thus, according to the model a very polar solvent, such as water, does not necessary yield a higher TPCS. Results from the few experiments that have been performed [18–20] confirm theoretical predictions [15,16] that the TPCS do not show a monotonic dependence on the polarity of the solvent. For distyrylbenzene chromophores [18], it was found that the TPCS in tetrahydrofuran is bigger than in water. However, using the TPA fluorescence method with femtosecond pulses for a series of fluorene and carbazole-core molecules [19], the maximum TPCS was for a solvent of intermediate polarity (acetophenone). While, in the TPA study of the novel dibenzothiophene core-branched structures [20], polar solvents resulted in the highest TPCS.

In this paper, we present our experimental observations on how solvents can vary the TPA and TPF properties of fluorescent rhodamine (Rh) dyes Rh6G, RhB and Rh101. Rhodamines are well-known xanthenes dyes, which have been extensively used for many widespread applications in single-molecule detection [24], DNA-sequence determination [25], fluorescence labelling [26], etc. due to their strong fluorescence over the visible spectral region. Molecular geometries of rhodamine dyes are well-known [27,28] and indicate that all the structures are non-centrosymmetric. In general, for centrosymmetric molecules, TPA is forbidden when tuned to the transitions at one-half of the excitation frequencies. However, for non-centrosymmetric molecules due to symmetry relaxations, the single-photon absorption (SPA) peaks and TPA peaks may coincide. So we set our primary aim to find the effect of solvent polarity on the correlation of SPA and TPA peaks for all the dyes.

We use the conventional open aperture (OA) z-scan experiments [29,30] as well as two-photon fluorescence (TPF) studies [31,32] for these dyes in different solvents to compare the results from the two techniques. We find that although the absolute values are different from the two studies due to calibration issues, our calculated Two-Photon Excited Action Cross-Section (TPEACS) spectra from TPF studies exactly match with the TPA spectra. We also discuss another useful concept of the two-photon figure-of-merit (*T*-factor or simply *T* = *βλ*/*n*_2_, *λ* = excitation wavelength, *β* = TPA coefficient, and *n*_2_ = nonlinear refractive index of the dye) [33,34], which quantify the nonlinear optical (NLO) efficiency ratio of the dyes to facilitate the search of suitable nonlinear materials in a particular wavelength range. We compare solvent-dependent *T*-factor for Rh6G and RhB dyes in the wavelength range of 750–810 nm.

## 2 Background theory

We derive, for calculation of *σ*_2_ by z-scan method [29]:

(1) $$\text{Transmitted}\;\text{power}=P(z,t)=P_{i}(t)e^{-\alpha L}\;\text{ln}\left[\frac{1+q_{0}(z,t)}{q_{0}(z,t)}\right]$$

where $q_{0}(z,t)=\beta I_{0}(t)L_{\text{eff}}/(1+(Z^{2}/Z_{0}^{2}))$; $P_{i}(t)=\pi w_{0}^{2}I_{0}(t)/2=\text{instantaneous}\;\text{input}\;\text{power}\;(\text{within}\;\text{the}\;\text{sample})$; *β* = two-photon absorption coefficient; *I*_0_(*t*) = intensity at the focus; *L*_eff_ = effective sample length = 1 − *e*^−*αL*^/*α*; *α* = linear absorption coefficient; *L* = sample thickness; $Z_{0}=\text{diffraction}\;\text{length}=\pi w_{0}^{2}/\lambda$; $w_{0}=\text{minimum}\;\text{beam}\;\text{radius}\;\text{at}\;\text{the}\;\text{focus}$; *λ* = wavelength.

For a temporally Gaussian pulse, (1) can be time integrated to give the normalized energy transmittance:

$$T(z)=\frac{1}{\sqrt{\pi }q_{0}(z,0)}\int_{-\infty }^{\infty }\text{ln}[1+q_{0}(z,0)e^{-\tau^{2}}]\;d\tau$$

For, |*q*_0_| < 1, this transmittance can be expressed in terms of the peak irradiance in a summation form:

(2) $$\begin{array}{ll} T(z) & =\sum_{m=0}^{\infty }\frac{{\left[-q_{0}(z,0)\right]}^{m}}{{\left(m+1\right)}^{3/2}}\\ \; & =1-\frac{\beta I_{0}L_{\text{eff}}}{2^{3/2}(1+(z^{2}/z_{0}^{2}))}+\text{Higher}\;\text{terms}\ldots \\ \; & =1-\frac{\beta I_{0}(1-e^{-\alpha L})}{2^{3/2}\alpha (1+(z^{2}/z_{0}^{2}))}\\ \; & =1-\frac{\beta I_{0}L}{2^{3/2}(1+(z^{2}/z_{0}^{2}))}\;[\text{if}\;\alpha <<1]. \end{array}$$

At different *z* positions *T* are calculated and since all the other parameters are known, *β* can be calculated easily. Subsequent to obtaining the values of *β*, the TPCS *σ*_2_ of the chromophore (in units of 1 GM = 10^−50^ cm^4^ s/photon molecule) is generated from the expression:

(3) $$\sigma_{2}=\frac{\beta h\nu \times 10^{3}}{Nc}$$

where *ν* is the frequency of the incident laser beam, *N* is Avogadro constant, and *c* is the concentration of the chromophores in respective solvents.

The nonlinear refractive index *n*_2_ is obtained from closed aperture z-scan measurements. To obtain the refractive nonlinearity in the presence of two-photon absorption, the closed aperture scan was first divided by the open aperture scan. The experimental data is fitted to the expression given by Sheik-Bahae et al. [29], which relates the normalized transmittance *T*(*z*, Δ*Φ*_0_) directly to phase change Δ*Φ*_0_:

(4) $$T(z,\Delta \Phi_{0})\approx \frac{1+4\;\Delta \Phi_{0}x}{\left(1+x^{2})(9+x^{2}\right)}$$

where *x* = *z*/*z*_0_; Δ*Φ*_0_ = phase change = 2π*n*_2_*L*_eff_/*λ*; effective sample length =*L*_eff_ = [1 − exp(−*α*_0_*L*)]/*α*_0_, *α*_0_ = linear absorption and *L* = sample length. *n*_2_ is calculated from the theoretical fit to the above equation. Notably, the transmittance derived in the equation is actually for on-axis transmission and the expression is valid for a vanishingly small aperture. However for a non-zero aperture (20% in our case), the equation still works except for the fact that the sensitivity of the z-scan reduces. According to Sheik-Bahae et al. [29], the plot of “difference between normalized peak and valley transmittance (Δ*T*_p-v_) against |Δ*Φ*_0_| for various aperture sizes” shows, for all aperture sizes Δ*T*_p-v_ is found to be almost linearly dependent on |Δ*Φ*_0_|. However, the slope increases as we reduce the aperture size gradually, indicative of higher sensitivity with reduced aperture size. Our chosen aperture size of 20% transmission provides a balance between the sensitivity [29] as well as the signal to noise.

In the TPF based approach to determine TPCS, we measure the fluorescence signal generated by TPA. We can determine the TPEACS from the two-photon fluorescence signal. This TPEACS is linearly proportional to the TPCS *σ*_2_, with a constant of proportionality, which is the fluorescence quantum efficiency of the fluorophore [32]:

(5) $$\sigma_{\text{TPE}}=\sigma_{2}\eta_{2}$$

Fluorescence signals from new fluorophores (which are to be measured) are calibrated with respect to the fluorescence of a calibration sample. Thus, the ratio of these fluorescence signals determines values of TPEACS for new fluorophores. The ratio of the experimentally measured fluorescence signals is as follows [32]:

(6) $$\frac{{\langle F(t)\rangle }_{\text{cal}}}{{\langle F(t)\rangle }_{\text{new}}}=\frac{(\phi_{\text{cal}}\eta_{2\text{cal}}\sigma_{2\text{cal}}c_{\text{cal}}{\langle p_{\text{cal}}(t)\rangle }^{2})n_{\text{cal}}}{(\phi_{\text{new}}\eta_{2\text{new}}\sigma_{2\text{new}}c_{\text{new}}{\langle p_{\text{new}}(t)\rangle }^{2})n_{\text{new}}}$$

where 〈*p*_cal_(*t*)〉 and 〈*p*_new_(*t*)〉 are the incident powers for the calibration and the new fluorophores, respectively. The TPEACS of a new molecular fluorophore is then related to known experimental wavelength-dependent parameters, including the TPEACS of the calibration standard, as described by

(7) ση2new2new=ϕcalη2calσ2cal(λ)ccalϕnewcnew〈pcal(t)〉2〈pnew(t)〉2〈F(t)〉new〈F(t)〉calncalnnew

For all our z-scan and TPF experiments, we take the known value of *σ*_2_ for rhodamine-6G in MeOH at 806 nm as the reference [30] for calibrating our measurement technique.

## 3 Materials and methods

Dyes used, Rh6G (dye content: 99%), RhB (dye content: 99%), and Rh101 (dye content: 99%) are purchased from Sigma–Aldrich, and are used without further purification. Purity of the dyes is a very important factor, as impurities can severely affect their photophysics through competitive processes. However, with the given dye purity, such effects can only add an error-bar of at most a few percent in our experimental data. All the solvents used were of spectra-grade quality. Linear absorption spectra for all rhodamine dyes in various solvents at 10^−5^ M concentration are measured in 1 cm long quartz cuvette with a diode-array spectrophotometer (Agilent-8453), after subtracting the cuvette and the respective solvent contribution. However, for all the nonlinear measurements, a concentration of 0.005 M is used.

**Fig. 1:**
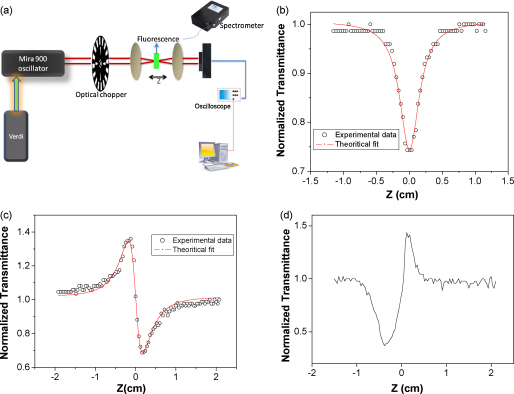
(a) Femtosecond experimental setup. (b) Open aperture z-scan trace of Rh6G in MeOH. (c) Closed aperture z-scan plot of Rh6G in MeOH at 806 nm by MHz oscillator showing negative *n*_2_. (d) Closed aperture z-scan plot of Rh6G in MeOH at 810 nm by kHz laser Odin, showing positive *n*_2_.

TPCS values of the samples are measured by open aperture z-scan (intensity scan) and two-photon induced fluorescence methods in the same experimental setup. Our femtosecond experimental setup (Fig. 1(a)) involves mode-locked high repetition rate (HRR) Coherent Mira Ti:sapphire laser (Model 900) which is pumped by Coherent Verdi frequency doubled Nd:vanadate laser. The model 900 Mira is tunable from 730 to 900 nm and its repetition rate is 76 MHz. We employ blanking in our nonlinear transmission measurements by using a mechanical chopper MC1000A from Thorlabs at 50% duty cycle. The optimization of the frequency of the chopper is performed by eliminating the cumulative thermal effect arising from sample heating by HRR pulses at excitation wavelength [35,36]. We also used 1 kHz amplified laser system (Odin, Quantronix Inc.) for a single wavelength TPF experiments of Rh dyes without chopper at 810 nm. Liquid samples are held in a 1 mm path length quartz cuvette for OA z-scan experiments. Using a 20 cm focal length lens, the pulses are focused into the sample cell, which resulted in ∼1 GW/cm^2^ intensity at the focus. Gaussian beams were used for all the experiments without any spatial filtering. The 1 mm long sample cell satisfies the condition that the cell-length is less than the Rayleigh range of the focusing lens. Rayleigh range in our setup is 1.2 mm at 770 nm, since the beam waist at focus is 17 μm. We scanned the sample through the focal point of the lens using a motorized translation stage (Newport Inc. model ESP 300), which has a minimum step-size of 0.1 μm. This allows for a smooth intensity scan of the sample. The transmitted beam through the sample is focused with a 7.5 cm focal length lens into a UV-enhanced amplified silicon photo detector (Thorlabs DET210). The peak-to-peak value from the photodiode is measured with an oscilloscope (LeCroy WaveRunner Model LR6100R), which is triggered by the chopper frequency. The delay stage and the oscilloscope are interfaced with the computer using a GPIB card (National Instruments Inc.) and the data is acquired using LabVIEW programming. Closed aperture z-scan experiments are also performed to calculate *n*_2_ by 20% opening of the aperture in the far field, by keeping all the other experimental parameters intact. Fig. 1(b) and (c) show the representative open and closed aperture (CA) z-scan plots of Rh6G in MeOH at 806 nm. Both Rh6G and RhB shows negative *n*_2_ in all the solvents with a characteristic pre-focal transmittance maximum (peak) followed by a post-focal transmittance minimum (valley) from CA z-scan experiments from the HRR laser. The highest value of *n*_2_ is obtained from RhB in CHCl_3_ as −3.5 × 10^−13^ cm^2^/W. We also measured nonlinearity of the neat solvents used in the experiments with both the closed and open aperture z scans and found that except for MeOH, all the other solvents have no measurable nonlinearity at identical laser powers and same experimental conditions. Recently, we have shown that alcohols, including MeOH, have negative *n*_2_ values [37] when measured with HRR lasers. We also measured *n*_2_ values with our kHz amplified laser (Odin), which is pumped by the Coherent Nd–YAG 120 ns Corona laser. Interestingly, we always found positive *n*_2_ for all the materials that we have studied with the amplified laser, i.e., a characteristic pre-focal transmittance minimum (valley) followed by a post-focal transmittance maximum (peak) (Fig. 1(d)) with kHz laser. In fact, very low average laser power from the amplified laser also generated positive *n*_2_. Experiments performed by changing the laser repetition rate using 120 ns Corona pulses at 532 nm, whose repetition rate was changed from 1 to 25 kHz, however, show negative *n*_2_ values just like the measurements from the HRR lasers. Thus, we conjecture that the sign of *n*_2_ is a peak intensity dependent phenomenon as the peak power for the amplified laser is 100 times higher than the oscillator. Under our experimental conditions, the laser repetition rate does not affect the sign of *n*_2_. To avoid such peak intensity dependent effects, we have performed all the *n*_2_ measurements using the Ti:sapphire HRR laser only. After we determined *β* and *n*_2_, we calculated the two-photon figure-of-merit (*T*-factor) for these chromophores in different solvents and wavelengths.

The two-photon fluorescence signal is generated with the same above-mentioned setup with a stationary 1 cm fluorescence cuvette placed at the focal point of the lens. TPF signal is collected at right-angles through the fiber-tip of the CCD based Ocean-Optics monochromator (HR-2000). The fluorescence signal to the monochromator is filtered through an IR filter.

## 4 Results and discussion

### 4.1 TPA spectra

**Fig. 2:**
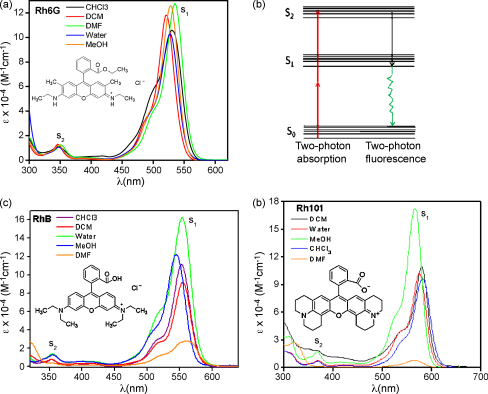
Linear absorption spectra of the rhodamine dyes in different solvent medium: (a) Rh6G, (c) RhB and (d) Rh101 indicating the S_1_ and S_2_ transition peaks. A concentration of 10^−5^ M is used for measuring the spectra. Corresponding dye structures are also given in the inset. (b) The Jablonski diagram of rhodamine dyes.

As seen in Fig. 2, the one-photon absorption spectra of all the dyes are similar. The lowest energy absorption band consists of a large peak with a small shoulder. The main absorption band is attributed to the S_1_ state and the shoulder around 498 nm for Rh6G indicates the presence of dimer species [38]. There is another small peak in the near-UV region (350–400 nm). This is assigned as S_2_ state [39] for these dyes. The Jablonski diagram of rhodamine dyes is given in Fig. 2(b). In our laser excitation range of 730–900 nm, there is no single-photon absorption for the dye. Table 1 summarizes the linear absorption data of all the dyes in different solvents. However, as the increase in the electron delocalization among the dyes follows the order: Rh101 > RhB > Rh6G, the UV–vis peaks of Rh101 and RhB get red-shifted compared to Rh6G peaks. We find that irrespective of solvents, the UV–vis peaks for Rh101 are maximally red-shifted.

**Fig. 3:**
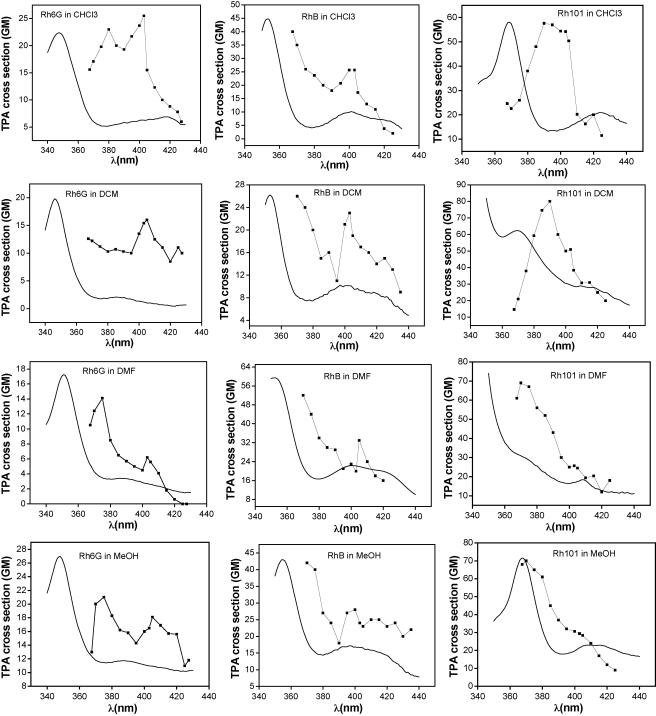
Comparison of one-photon (solid lines) and two-photon (lines + symbol) absorption spectra of rhodamine 6G, rhodamine B and rhodamine 101. The *y*-axis values represent two-photon absorption cross-sections for the dye. The one-photon intensities are plotted in arbitrary scale at the same *y*-axis. At the *x*-axis, the TPA spectra are plotted against half the excitation wavelength value to allow comparison of the transition wavelengths for the one-photon and two-photon allowed electronic transitions.

The improvements in laser systems in the recent years have enabled various groups to measure TPA spectra for rhodamine dyes [40,41]. We also see similar features in our measured TPA spectra in the wavelength range of 740–860 nm (Fig. 3). The TPA peaks are more intense in the blue region of the excitation wavelengths and in all the TPA spectra TPA intensity diminishes rapidly after 840 nm. However, our main interest is to investigate how the TPA peaks due to S_0_–S_2_ transition changes in the three different dyes and also in different solvent media for each of the dyes.

TPA spectra of Rh6G (Fig. 3) actually follow the SPA spectra and consist of two peaks in all the solvents. But the peaks are red-shifted as compared to the SPA spectra. We pointed out earlier that, for centrosymmetric molecules, TPA is forbidden when tuned to the transitions at one-half of the excitation frequencies. However, for non-centrosymmetric molecules due to symmetry relaxations, the single-photon absorption (SPA) peaks and TPA peaks may coincide. So for these non-centrosymmetric rhodamine dyes, we assume, that the lower energy TPA peak corresponds to the very weak SPA maxima at 400 nm region and the higher energy TPA peak correspond to 350 nm S_2_ peak. We find, the relative intensity of the TPA peaks in nonpolar solvents CHCl_3_ and DCM are very different from the corresponding one-photon spectra at half of the excitation wavelength, because transition falling at 400 nm region is much weaker than the one at 350 nm, whereas the opposite is true for the TPA spectra. One-photon pre-resonance effect [42] cannot explain these phenomena. However, in polar solvents, the relative intensities of the TPA peaks follow same trend as the SPA spectra. Possible explanation for this phenomenon can be that the weak single-photon peaks at 400 nm may be due to the vibronic sub-bands coupled with electronic states. The coupling of the odd parity component in the electronic state with odd parity component of vibrational state may give rise to even parity component [41], which can be accessible from ground state via two-photon transition. In nonpolar solvents this efficient vibronic coupling may be responsible for the low energy but higher intense TPA peaks. As excitation energy increases, this overlap diminishes due to the energy matching constraints, and the final excited state character approaches the real two-photon allowed S_2_ state. Previous theoretical [43,44] and experimental [45,46] studies also confirm vibronic induced coupling as a very important factor to be considered during TPA process. Two very important observations from these studies are: Firstly, one- and two-photon excitations may reach the same excited state due to relaxation of symmetry rules via vibronic coupling in the multibranched structures and secondly, the TPA spectra are always ‘structured’ when there is vibronic coupling involved. We also see similar features in the TPA spectra of Rh6G. In polar solvents MeOH and DMF, however, the ground electronic states are more stable than the excited states and a perfect overlap condition is difficult to find as compared to the nonpolar solvent case. Thus the solvents change the TPA properties of these chromophores through a change in the dielectric constant of the medium, which in turn can change the chromophore electronic structure; or by inducing geometrical distortions like a change in the torsional angle between the aromatic rings of the chromophores. However, it is not possible to specifically identify how the solvent is affecting the vibronic coupling mediated TPA process here.

RhB has a slightly different molecular geometry than Rh6G and its overall symmetry will be lower due to the removal of one ethyl group from the Rh6G structure. In RhB, the above-mentioned effect is less evident as we see lower energy TPA peaks are not higher in intensity (Fig. 3), but only comparable to the higher energy peaks only. However, the TPA peaks in RhB coincide with the one-photon peaks unlikely of Rh6G.

TPA spectra of Rh101 (Fig. 3) are completely different than the other two dyes. They consist of one peak in their TPA spectra. The molecular structure of Rh101 is largely different than that of Rh6G and RhB. Compared to Rh6G and RhB, Rh101 has less freedom for rotation due to the inclusion of the N-atoms in the rigid ring structure. We now may infer that structures or multiple peaks in the TPA spectra are due to the additional ethyl and methyl groups present in Rh6G and RhB, which are responsible for solvent assisted efficient vibronic coupling. But in Rh101 the methyl and ethyl substituents are absent and may be that is the probable reason of single peak in TPA spectra of the dye. Interestingly enough, with nonpolar solvents CHCl_3_ and DCM the TPA peaks do not coincide with SPA S_2_ peak. The peaks are clearly red-shifted. But with the MeOH and DMF solvents the TPA peak actually coincide with the S_2_ peak indicating polar solvents actually assist the two-photon absorption process to reach the pure two-photon allowed state in this particular case.

We are not able to measure any TPA in water for all the dyes under our experimental conditions by OA z-scan, though it has a quite high single-photon absorption coefficient (*ɛ* = 10, 2800 M^−1^ cm^−1^). This is due to the strong hydrogen bonding capabilities of water. For Rh6G, CHCl_3_ produces highest *σ*_2_ (26 GM) at 806 nm. For RhB, the highest *σ*_2_ (53 GM) is obtained with DMF, at 740 nm. Highest *σ*_2_ is obtained with Rh101 among the three dyes (80 GM) in the nonpolar DCM solvent at 780 nm. Table 2 summarizes all the TPA peaks of the dyes in different solvents.

There are two possible explanations why Rh101 gives highest *σ*_2_, simply from the molecular structure correlation (Fig. 2). Firstly, electron delocalization in the dye structures, which facilitates the TPA transition increases in the order: Rh101 > RhB > Rh6G and hence, we get the highest *σ*_2_ for Rh101. Secondly, all these dyes can have internal rotations in the excited states due to their flexible structures. Internal rotations within the molecule can lead to many nonradiative decay channels, which result in a loss of energy and as such in lowering the TPA process. Recently Zheng et al. have shown [47] increased TPEACS value for a conformationally restricted dipyrromethene boron difluoride (BODIPY) dye, having a naphthalene moiety at meso position, compared with the same dye having phenyl group at the meso position. This makes it suitable for free internal rotation. They show that the lower TPEACS value for the dye having phenyl moiety is justified, as the internal rotation causes a loss of energy from nonradiative molecular relaxation of the excited state for the chromophore. Similarly in our case, compared to Rh6G and RhB, Rh101 has less freedom for rotation due to the inclusion of the N atoms in the rigid ring structure. As a result of this suppressed internal rotation in Rh101, we register higher *σ*_2_ value. And about the role of solvent in modulating the absolute TPA cross-section, it is not clear enough as we mentioned earlier also. The distinct case of water as a solvent which does not show any results from either the open or closed aperture z-scan experiments can perhaps be understood if we consider that water can influence the electronic structure of chromophores via hydrogen bonding and a corresponding decrease in the TPA.

**Fig. 4:**
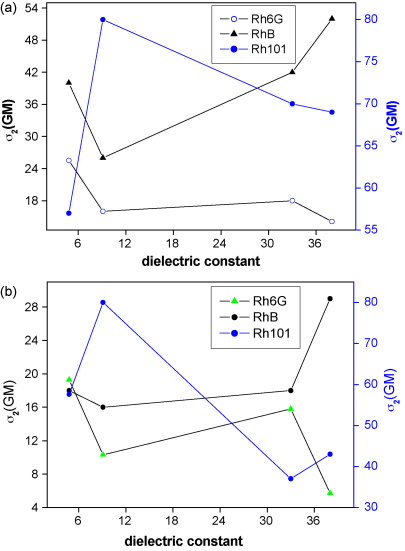
(a) Maximum TPA cross-sections among all measured wavelengths for the different dyes as a function of solvent polarity. (b) Maximum TPA cross-sections of the dyes as a function of solvent polarity at 780 nm excitation. In both the graphs TPA cross-sections of Rh101 are plotted in a different *Y*-scale in the right side.

The variation in the maxima value of TPCS of the dye molecules with the polarity of the solvent irrespective of the excitation wavelength is shown in Fig. 4(a). We find that there is no exact correlation between *σ*_2_ and the solvent dielectric constant. The highest *σ*_2_ values for Rh6G and Rh101 dyes are observed in the nonpolar solvents, CHCl_3_ and DCM respectively, while for RhB the highest *σ*_2_ is in case of the polar DMF solvent. In Fig. 4(b) we plot maximum *σ*_2_ against solvent polarity at 780 nm, and again an exact similar trend is observed. Overall, nonpolar solvents are better than polar solvents for TPA process. So, a general rule of thumb for the dependence of TPA cross-sections on solvent polarity cannot be extracted accurately. However, we should also note that such a solvent polarity dependent rule might not exist, simply because several other factors, such as, solute–solvent interactions, chromophore characteristics like solubility differences, etc. might be playing very important roles and may need to be considered for arriving at any general conclusion. Low solubility and hydrophobic interactions can also lead to aggregation or structure changes with a corresponding modification of linear and nonlinear optical responses [48,49].

### 4.2 Two-photon fluorescence study

**Fig. 5:**
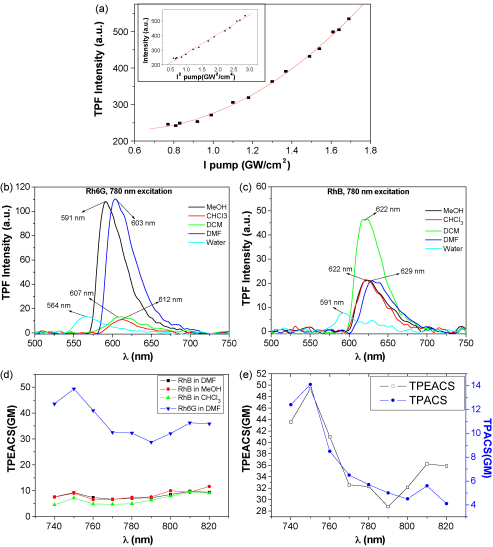
(a) Plot of two-photon fluorescence (TPF) intensity for Rh6G in MeOH versus excitation intensity of the laser showing quadratic dependence. Inset shows the linear dependence of TPF intensity on the square of the excitation intensity. (b and c) TPF spectra of Rh6G and RhB, respectively in different solvents at 780 nm excitation. (d) Calculated TPEACS spectra of different dyes in different solvents. (e) Comparison of TPA and TPEACS spectra of Rh6G in DMF measured from z-scan and TPF technique, respectively.

We present here two-photon fluorescence studies of the dye solutions in different solvents which emit upconverted fluorescence signals when excited with the near IR laser pulses of MIRA. Fig. 5(a) shows the quadratic dependence of the fluorescence intensity when plotted with respect to their average excitation laser powers at 810 nm. When combined with absorption spectra, this quadratic dependence provides reliable evidence that the fluorescence emission originates from the TPA process. Fig. 5(b) shows the TPF spectra of Rh6G in different solvents, when excited at 810 nm. The interactions between solvent and dyes affect the energy states between the ground and excited states. As emission always occurs from a lower-lying state (Fig. 4), spectral shift towards longer wavelength is seen. Solvent-dependent spectral shifts are generally described by the Lippert–Mataga equation [22]:

(8) $$\nu_{\text{A}}-\nu_{\text{F}}=\frac{2}{hc}\left(\frac{\varepsilon -1}{2\varepsilon +1}-\frac{n^{2}-1}{2n^{2}+1}\right)\frac{{\left(\mu_{\text{E}}-\mu_{\text{G}}\right)}^{2}}{a^{3}}$$

where *h* = Planck's constant, *c* = velocity of light, and *a* = radius of the cavity in which the fluorophore resides. *ν*_A_, *ν*_F_ are the absorption and emission maximum in wave number unit. *ɛ* and *n* are dielectric constant and refractive index of the solvent. We find, the peaks of the TPF spectra are red-shifted from their linear absorption maximum (as shown in Table 1) to 591 nm, 603 nm, 607 nm and 612 nm for their respective solvents: MeOH, DMF, DCM and CHCl_3_. For Rh6G, polar MeOH and DMF are the two solvents which show significant TPF intensity while, nonpolar DCM and CHCl_3_ show very less TPF intensity.

Now, TPF spectra of RhB in different solvents are shown in Fig. 5(c). Correspondingly, the TPF peaks are red-shifted from their linear absorption maximum, when excited at 810 nm. But unlike Rh6G, the TPF peaks for RhB actually coincides at 620–622 nm for all the solvents except for DMF, wherein there was a 7 nm red-shift to 629 nm. For RhB, TPF intensity is maximum in nonpolar DCM solvent. In general, however, solutions with polar solvents show more red-shift in the fluorescence spectra than its nonpolar counterparts. The disagreement from the above generalization in the TPF spectral shifts for RhB can be understood on the basis that the generalization is in fact an approximation that is devoid of several factors, such as, the effect of hydrogen bonding, viscosity of solvents, consideration of dipole moment and density of solvents, solubility of the dyes, etc. Unusual behavior seen may be due to presence of more than one type of interactions.

TPF spectra of Rh101 are not shown, as in most solvents the TPF intensities of this dye are very low. The TPF data in water solutions for both the dyes require a special mention, since we had to use a dye concentration of 10^−4^ M owing to the low solubility of these dyes in water. However, at such a low concentration, the TPF signal is extremely small and we had to double the excitation power for the TPF spectra reported in Fig. 5(b) and (c). However, we got some quantitative measure of the nonlinearity of these dyes in water, as *σ*_2_ value calculated from TPF technique for Rh6G and RhB in water are 8 and 12 GM, respectively at 810 nm. The blue-shift in the peak positions for the Rh dyes in water is an effect of dilution. The red-shift with higher chromophore concentration is attributed to the reabsorption of shorter wavelengths in the emission spectra.

We also repeated all the TPF experiments for all the dyes with our kHz laser, Odin, but only at a fixed wavelength of 810 nm, keeping all the other experimental parameters unchanged. Such an experimental setup has minimal thermal effects as compared to the HRR laser experiments. Our aim in doing so has been to make a comparison of the TPCS or TPEACS values calculated from the TPF data with MHz oscillator, MIRA, at the same wavelength, where thermal effects are minimized by blanking the excitation with an optical chopper [35,36]. Table 3 gives a summary of all the TPF data of Rh dyes measured by both Mira and Odin at 810 nm. The main anomaly in this set of data is from the calculated TPEACS values of the dyes in DMF solvent from Mira and Odin. For the experiments performed with kHz Odin, we have used 10^−4^ M concentrations of the dyes for measurement. At such low concentrations (10^−4^ M), the dyes in DMF solvent result in very low TPF signal and correspondingly a low TPEACS value. It is, in fact, difficult to provide any better reasoning.

Finally, Fig. 5(d) shows the TPEACS spectra for some of the Rh dyes in different solvents. The total fluorescence intensity was obtained by integrating the area under each TPF spectrum using a fixed baseline. Barring the absolute value, the TPEACS spectra almost exactly maps the TPA spectra generated from z-scan studies (Fig. 5(e)). This emphasizes the sensitivity and accuracy of our technique.

### 4.3 Two-photon figure-of-merit

The two-photon figure-of-merit or the *T*-factor for a particular nonlinear optical material is an unitless parameter, which is useful for applications in all-optical switching [50], where the essential requirement is *T* < 1. We report here such a database of *T*-factor for Rh6G and RhB dyes at different wavelengths at a concentration of 10^−3^ M (Table 4). For both the dyes in our experiments, *T* < 1 in all the solvents used. Overall, *T* values reach a maximum in DMF solvent for both the dyes. Interestingly, we also find that DMF is the solvent which gives lowest *n*_2_ irrespective of the dyes and wavelengths. Refractive index is a high frequency response and depends on the instantaneous motion of the electrons within the solvent molecules, which occurs during light absorption [22]. This motion of the electrons is hindered if the viscosity of a particular solvent is high. Probably, this is why we get lowest *n*_2_ values in case of DMF as its viscosity is the second highest among all the solvents, after water (Table 5). Water is the most viscous among all the solvents reported here and as such there are no CA z-scan experimental data possible for water in the case of all the dyes in our experiments. Earlier, in Section 4.1, we have referred to the dimerization of rhodamine at moderate dye concentrations. From Fig. 3, it is evident that between Rh6G and RhB, RhB probably has more tendency to form dimer than Rh6G, since shoulders in the absorption spectra are more evident for RhB than Rh6G. Dimer formation suppresses TPF [38], as a result the rest of the energy is deposited as thermal energy which may also increase the *n*_2_ of RhB as compared to that of Rh6G.

## 5 Conclusions

We have demonstrated elaborately how solvents play an important role in modulating the vibronic coupling mediated TPA process and also relative intensities, peak positions in the TPA spectra of rhodamine dyes. However, a quantitative description of the solvent effect on TPA and TPF is perhaps the most challenging task for a spectroscopist due to the unavailability of a particular model which accommodates all possible circumstances. Highest *σ*_2_ is obtained with nonpolar DCM solvent for Rh101. We have generally shown that the solvent polarity does affect the linear and nonlinear properties of the chromophores. However, other factors, such as, hydrogen bonding, viscosity, solute–solvent interactions, and solubility, should also be looked into for better solving this complex problem. A useful figure-of-merit database is also presented for Rh6G and RhB dyes, in relevance with third-order nonlinear applications. Finally and most importantly, our technique shows that calculated TPEACS spectra for the dyes exactly match the TPA spectra.

## Figures and Tables

**Table 1:** Summary of linear absorption data of the rhodamine dyes in different solvents. All the dyes show two absorption peaks and the data shown are corresponding $\lambda_{\text{max}}^{\text{abs}}$ (nm) and those in the parenthesis represent their corresponding absorption coefficient, *ɛ* (×10^−4^ M^−1^ cm^−1^). Instead of a peak there is a shoulder at the short wavelength region for Rh101 in DMF and hence it is not mentioned.

Dyes	MeOH	DCM	CHCl_3_	DMF	Water
Rh6G	348 (1.9)	346 (1.3)	347 (1.1)	351 (1.2)	347 (1.1)
	528 (13.23)	521 (11.82)	530 (10.6)	535 (12.77)	528 (10.28)

RhB	355 (1.2)	353 (0.8)	353 (0.8)	352 (0.3)	354 (1.3)
	545 (12.23)	555 (9.18)	550 (10.85)	560 (2.72)	553 (16.26)

Rh101	367 (1.7)	366 (1.9)	369 (0.7)	570 (0.38)	368 (0.8)
	565 (17.3)	580 (10.9)	582 (9.65)		575 (10.21)

**Table 2:** Summary of two-photon absorption data of the rhodamine dye in different solvents. Data shown are corresponding *λ*_TPA-max_(s) in nm and those in the parenthesis represent their corresponding two-photon absorption cross-section values in GM unit.

Dyes	MeOH	DCM	CHCl_3_	DMF
Rh6G	750 (21)	735 (13)	760 (23)	750 (14)
	810 (18)	810 (16)	806 (26)	806 (6)
		850 (16)		

RhB	740 (42)	740 (26)	735 (40)	740 (52)
	800 (28)	806 (23)	800 (26)	810 (33)

Rh101	740 (70)	780 (80)	780 (58)	740 (69)

**Table 3:** Comparison of the TPEACS and few TPCS values of Rh dyes measured by both MHz oscillator Mira and kHz amplifier Odin at 810 nm. The absent values in the TPEACS column are due to either very low TPF signal or cannot be done due to solubility constraints.

Dyes	Solvent	TPEACS (GM)		Quantum yield (if known)	*σ*_2_ (GM)	
		Odin	Mira		Odin	Mira
Rh6G	DCM	18	–			
	DMF	10	36			
	CHCl_3_	9	–			
	H_2_O	10.3	7.15	0.95	10.6	7.5

RhB	MeOH	9	9	0.63	15	15
	DCM	18	14			
	DMF	–	10			
	CHCl_3_	7	9.48			
	H_2_O	4	3.6	0.31	13	12

Rh101	MeOH	15	11	1	15	11
	DCM	6	–			
	DMF	1	–			
	CHCl_3_	9	–			
	H_2_O	3	1.6			

**Table 4:** Figure-of-merit database of Rh6G and RhB in different solvents at different wavelengths (in nm) of MIRA oscillator. The values of *β* are given in units of cm/GW (GW = Gigawatt). All the *n*_2_ values are negative and for convenience it is given as: −*n*_2_ × 10^13^ cm^2^/W. Figure-of-merit (*T*) is unitless.

Dyes	Solvent	Wavelength	*β*	−*n*_2_	*T*
Rh6G	MeOH	750	0.4773	1.6	0.223
		780	0.3735	1.278	0.228
		806	0.403	1.7873	0.182

	CHCl_3_	750	0.45	2.5325	0.133
		780	0.4561	2.3505	0.151
		806	0.6228	2.5603	0.196

	DCM	750	0.2545	1.0913	0.175
		780	0.2435	1.1776	0.161
		806	0.3761	1.2067	0.251

	DMF	750	0.3205	0.8158	0.294
		780	0.1347	0.5969	0.176
		806	0.1514	0.5742	0.213

RhB	MeOH	750	0.9091	3.0122	0.227
		780	0.4255	2.5053	0.132
		806	0.5862	2.612	0.181

	CHCl_3_	750	0.5909	3.577	0.124
		780	0.4255	3.3676	0.099
		806	0.6277	3.1166	0.162

	DCM	750	0.5455	3.4435	0.119
		780	0.3782	2.9062	0.102
		806	0.5612	2.7932	0.162

	DMF	750	1.0	2.9388	0.255
		780	0.6855	2.435	0.22
		806	0.4885	2.155	0.1827

**Table 5:** Few physical properties of the solvents, used in the experiments.

Solvent	Dieletric constant	Dipole moment, *D*	Viscosity (×10^−3^ Pa s)	Density (g/ml)
Chloroform	4.8	1.01	0.54	1.4799
Dichloromethane	8.9	1.6	0.423	1.3168
Dimethylformamide	37	3.8	0.796	0.9455
Methanol	32.6	1.6	0.5455	0.7866
Water	78.39	1.84	0.8905	0.997
